# Retraction Note: The analysis of boric acid effect on epithelial-mesenchymal transition of CD133 + CD117 + lung cancer stem cells

**DOI:** 10.1007/s00210-024-03613-7

**Published:** 2024-11-11

**Authors:** Tuğba Semerci Sevimli, Murat Sevimli, Aynaz Ghorbani, Varol Şahintürk, Emilia Qomi Ekenel, Tuğba Ertem, Bahar Demir Cevizlidere, Burcugül Altuğ, Özlem Tomsuk, Onur Uysal, Sibel Güneş Bağış, Hüseyin Avci, Fatih Çemrek, Zarifa Ahmadova

**Affiliations:** 1 Stem Cell, Cellular Therapy, and Stem Cell Production Application and Research Center (ESTEM), Eskisehir Osmangazi University, 26040 Eskisehir, Turkey; 2 Department of Stem Cell, Institute of Health Sciences, Eskisehir Osmangazi University, 26040 Eskisehir, Turkey; 3 Department of Histology and Embryology, Faculty of Medicine, Eskisehir Osmangazi University, 26040 Eskisehir, Turkey; 4 Graduate School of İnformatics, Middle East Technical University, Ankara, Turkey; 5 Department of Metallurgical and Materials Engineering, Eskisehir Osmangazi University, 26040 Eskisehir, Turkey; 6 Department of Statistics, Faculty of Science and Letters, Eskisehir Osmangazi University, 26040 Eskisehir, Turkey

**Retraction Note: Naunyn-Schmiedeberg's Archives of Pharmacology (2024) 397:6791-6802**

10.1007/s00210-024-03062-2

The Editor-in-Chief has retracted this article. After publication, concerned were raised about potential image duplications within Figure 4. The original images for Figure 4 could not be recovered in order to verify the correct panels. The Editor has therefore lost confidence in the underlying data and conclusions of the article.

Authors Tuğba Semerci Sevimli, Murat Sevimli, Hüseyin Avci and Varol Şahintürk agree with this retraction. All other Authors did not respond to correspondence from the Publisher about this retraction.

